# The mediating role of English learning motivation between socioeconomic status and pragmatic awareness

**DOI:** 10.3389/fpsyg.2024.1471108

**Published:** 2025-01-08

**Authors:** Xiaomeng Hui, Yuqing Chen

**Affiliations:** ^1^College of Foreign Studies, Northeastern University, Shenyang, China; ^2^School of Foreign Languages, Southeast University, Nanjing, China

**Keywords:** L2 motivational self-system (L2MSS), pragmatic awareness (PA), socioeconomic status (SES), motivation, ideal L2 self

## Abstract

Socioeconomic status (SES) has received great attention in learning a second or foreign language (SL/FL). However, little research has investigated the association between SES and SL/FL pragmatic learning, let alone the influencing pathways of SES on SL/FL pragmatic awareness (PA). Therefore, this research aimed to address the link between learners’ SES and PA with the mediating effects of learning motivation based on the L2 motivation self-system (L2MSS) theory by surveying 292 Chinese EFL university students. Structural equation modeling analyses indicated that: (1) SES had no significant effect on ought-to L2 self and intended learning efforts; (2) ought-to L2 self and intended learning efforts had significant predictive effects on PA; (3) SES positively and directly predicted EFL learners’ PA; and (4) ideal L2 self, attitudes toward L2 community, and attitudes toward learning English mediated the relationship between SES and Chinese EFL learners’ PA significantly.

## 1 Introduction

The importance of pragmatic awareness (PA) has been increasingly acknowledged in recent years as it is an important part of pragmatic competence (Ren, 2015; Yang and Ren, 2020), which in turn determines the success or failure of human interaction (García-Gómez, 2022; Yang, 2022). The development of foreign or second language pragmatic awareness requires the support of motivation to assist students in noticing target pragmatic norms and recognizing pragmatic inappropriateness or errors of an utterance (Takahashi, 2005; Chiravate, 2012; Yamato et al., 2013; Yang and Ren, 2020). Yang and Ren (2020) elaborated that students’ L2 motivation influences their pragmatic awareness, while the potential effect of motivation on pragmatic awareness in SLA has been largely underrepresented (Taguchi and Roever, 2017; Yang and Ren, 2020). Furthermore, as the antecedent of motivation, socioeconomic status (SES) has been demonstrated to play a prominent role in second/foreign language (SL/FL) learning. That is, socioeconomically privileged students tend to have more access to learning environments and resources (Butler, 2014; Ghorbani and Golparvar, 2020), gain more effective language input (Huang et al., 2018), are more willing to make efforts (Shin and So, 2018), display a higher level of motivation (Lee and Lee, 2023) and self-efficacy (Kormos and Kiddle, 2013), are more adapted to autonomous language learning with technology (Ghorbani and Golparvar, 2020), and use more cognitive, meta-cognitive, compensatory, and social strategies (Shin and So, 2018) in SL/FL learning.

Compared with research on influencing factors of pragmatic awareness in viewing learners as a homogeneous group, research focusing on the impact of individual differences on learners’ pragmatic awareness has gained great attention recently (Yang and Ren, 2020; Wang and Ren, 2023). SL/FL learning happens under the influence of a wider range of internal and external factors, such as learners’ motivation, learning environment, and learning resources (Oxford, 2016; Mercer, 2018; Paradowski and Jelińska, 2024), which are more accessible to learners from higher SES families (Butler, 2014; Ghorbani and Golparvar, 2020; Lee and Lee, 2023) and contribute to their SL/FL pragmatic awareness (Xu et al., 2009; Yang and Ren, 2020). Although it is widely accepted that SES is correlated with SL/FL pragmatic competence, much less is known about the pathways by which SES exerts its influence on pragmatic awareness in SL/FL learning.

Besides, research in the FL learning context has shown that the effect of SES on learning outcomes is indirect and mediated by other variables (e.g., Huang et al., 2018; Lee and Lee, 2023), calling to attach importance to the mediating role of motivation in SES and FL learning outcomes (Huang et al., 2018). Indeed, SES was discovered to have significant effects on English learners’ motivation (Lamb, 2012; Kormos and Kiddle, 2013; Butler, 2015; Butler and Le, 2018), which has been found to influence foreign language learning to notice of pragmatic strategy (Takahashi, 2005) and awareness (Chiravate, 2012; Yang and Ren, 2020). However, there is currently no research directly exploring the relationship between SES, motivation, and pragmatic awareness in foreign language learning contexts, disclosing a necessary field of investigation yet to be explored.

The current study aims to address these gaps by exploring the structural relations between SES and pragmatic awareness among Chinese EFL learners and investigating the possible mediating role of their motivation between SES and pragmatic awareness. The research questions are: (1) Does EFL students’ SES directly affect their motivation? (2) Is there a direct effect of EFL students’ motivation on their pragmatic awareness? (3) Is there a direct effect of EFL students’ SES on their pragmatic awareness? (4) Does EFL students’ learning motivation significantly mediate the relationship between their SES and pragmatic awareness? The present study is expected to expand the effect of SES on pragmatic awareness from the first language to the second or foreign language field and clarify the relationship between SES, motivation, and pragmatic awareness, as well as the mediating role of motivation variables. In addition, the present study can reveal how EFL students with different types of motivation (e.g., ideal L2 self vs. ought-to L2 self) handle their English pragmatic learning, which will provide information for English pragmatic pedagogy.

## 2 Literature review

### 2.1 SES and motivation

A student’s SES refers to an individual or a group’s ranking in social hierarchy according to some valued commodities they accessed, like wealth, power, and social status (Mueller and Parcel, 1981; Sirin, 2005), which is often operationalized as a combination of their parents’ educational background, parents’ occupation, and family income in educational research (e.g., Fan, 2011; Ensminger and Fothergill, 2014; Whitney and Bergin, 2018; OECD, 2019; Xu et al., 2021; Zheng and Mei, 2021; Ma et al., 2022). It has been widely recognized that SES plays an important role in students’ academic achievement (Pace et al., 2017; Nikolov and Csapó, 2018; Sanjurjo et al., 2018; Chen et al., 2021; Luo et al., 2021; Liu et al., 2023; Sanfo and Malgoubri, 2023), whereas research on the association between SES and students’ learning process and outcome in the FL context is quite recent (Butler and Le, 2018; Huang et al., 2018; Ghorbani and Golparvar, 2020). Most studies have showcased the positive role of students’ SES background in foreign language learning (e.g., Csapó and Nikolov, 2009; Butler and Le, 2018; Shin and So, 2018; Huang, 2022; Ma et al., 2022). Specifically, high SES contributes to improving students’ achievement in foreign language learning, which is achieved by influencing students’ language input (Huang et al., 2018), parental educational behavior (Butler, 2014), language learning with technology (Ghorbani and Golparvar, 2020), cognitive skills (Liu et al., 2020), and motivation (Lee and Lee, 2023). In summary, SES background positively contributes to the process and outcomes of foreign language learning, and the positive impact on outcomes is generally achieved indirectly through other factors such as motivation (Huang et al., 2018).

Language learning motivation greatly impacts SL/FL learning (Yang and Ren, 2020; Vonkova et al., 2021; Jia and Cheng, 2022). It has been widely acknowledged that learners with high levels of motivation perform better in second/foreign language acquisition than learners with low levels of motivation (Dörnyei, 2005; Papi, 2018; Gong et al., 2020; Sudina, 2021; Wang and Liu, 2022; Xu et al., 2022; Li and Han, 2024). Extensive research has explored the influencing factors of foreign language learning motivation, such as buoyancy (Jia and Cheng, 2022), SES (Kormos and Kiddle, 2013; Butler, 2015; Lee and Lee, 2023), self-efficacy (You et al., 2016), social support (Papi and Hiver, 2020; Trigueros et al., 2020; Jia and Cheng, 2022), and teaching approaches (Liu and Lan, 2016; Önal et al., 2019). Moreover, research has documented the predictive role of motivation in learners’ attitudes (Huong et al., 2017), emotions (Saito et al., 2018), willingness to communicate (Lin, 2019), engagement (Oga-Baldwin et al., 2017; Li and Han, 2024), and pragmatic awareness (Yang and Ren, 2020) in foreign language learning. However, the specific effect of motivation in the link between learners’ SES and pragmatic awareness in foreign language learning is still unclear.

The predictive role of learners’ socioeconomic background in foreign language learning motivation has been supported by previous studies (Muñoz, 2008; Lamb, 2012; Kormos and Kiddle, 2013; Butler, 2015, 2017; Shin and So, 2018; Lee and Lee, 2023). Among multiple motivational variables, the ideal L2 self has been investigated as a crucial motivational factor closely related to social class. Lamb (2012) concluded the ideal L2 self is an important factor only for the urban population. Specifically, due to the lack of role models or social contact with respected others, rural learners are less likely to develop strong possible self-images, resulting in holding less favorable views of their ideal selves than urban learners (Lamb, 2012; Kormos and Kiddle, 2013). Besides, empirical studies have reported a positive association between SES and English learning motivation among primary (e.g., Butler, 2015), secondary (e.g., Kormos and Kiddle, 2013), and college school students (e.g., Lee and Lee, 2023) and inferred that the impact of family resources on students’ English learning motivation varies depending on grades (Butler, 2015, 2017). Specifically, a higher family socioeconomic status is more conducive to the development of children’s motivation in English learning as the grade level increases. Hence, SES may have a strong influence on university students’ motivation to learn foreign languages.

*H1:* SES positively predicts EFL learners’ motivational variables.

### 2.2 L2 motivational self-system

Dörnyei (2005) L2 motivation self-system (L2MSS) is one of the leading theories widely used in the study of English learning motivation (Yousefi and Mahmoodi, 2022). This framework considers learners’ future self-images as the driving force behind their motivation. When learners perceive discrepancies between their present and future selves (i.e., ideal and ought-to selves), they are motivated to make efforts to bridge the gap. The L2MSS comprises three components: the ideal L2 self, the ought-to L2 self, and the L2 learning experience.

According to Dörnyei (2019), the ideal L2 self represents the learner as a proficient and skillful user of the target language, while the ought-to L2 self reflects external expectations, social or familial obligations, and the desire to avoid negative outcomes in language learning. Among these two kinds of selves, the ideal L2 is widely recognized as having higher predictive validity for L2 learning. Research has demonstrated that the ideal L2 self significantly and positively influences motivational intensity, persistence, intended efforts, and achievement in L2 learning (Al-Hoorie, 2018; Feng and Papi, 2020; Yousefi and Mahmoodi, 2022).

In contrast, while the ought-to L2 self has been found to be significantly and positively correlated with L2 motivational intensity (Feng and Papi, 2020) and intended effort (Al-Hoorie, 2018), it shows no significant correlation with L2 learning achievement (Al-Hoorie, 2018) and is negatively correlated with persistence in L2 learning (Feng and Papi, 2020).

The conflicting results regarding the ought-to L2 self may be explained by its nature. While meeting others’ expectations, avoiding negative outcomes, and fulfilling obligations can initially stimulate learners’ motivation and encourage their willingness to study (Papi et al., 2019; Feng and Papi, 2020), this effort may not necessarily translate into sustained or effective learning behavior.

On the other hand, learners driven by this kind of motivation aim to achieve the minimum goal of avoiding negative outcomes, resulting in a non-significant relationship with L2 achievement. Moreover, driven by this kind of motivation, learners are less likely to sustain long-term engagement and enthusiasm in L2 learning (Al-Hoorie, 2018). Thus, further exploration is needed to explore the impact of the L2 selves on specific L2 learning variables.

The present study identified L2MSS as the theoretical framework to explore the mediating role of motivation between Chinese EFL learners’ SES and pragmatic awareness firstly because it holds that L2 learners’ ideal and ought-to L2 self are constantly evolving and changing due to individual factors (e.g., learner’s socio-economic background and language proficiency) and environmental factors (e.g., instruction pattern, social background), which in turn stimulate or inhibit the formation and maintenance of motivation (Dörnyei, 2005), secondly because L2MSS has shown significant explanatory power in exploring the relationship between L2 motivation and multiple aspects of language learning achievement (Csizér and Gyula, 2017; Sasaki et al., 2017; Yang and Ren, 2020).

### 2.3 Motivation and PA

Pragmatic awareness has recently attracted considerable attention as it is an important part of pragmatic competence (Ren, 2015; Yang and Ren, 2020). It has been defined as “conscious, reflective, and explicit knowledge about pragmatics” (Alcón and Safont Jordà, 2008, p. 193). Bardovi-Harlig and Dörnyei (1998) pioneered the study of pragmatic awareness. The pragmatic awareness test they developed has been widely used by researchers to assess English learners’ noticing of pragmatic infelicities (Yang and Ren, 2020; Lv et al., 2021). Previous researchers have found that learning environment (Niezgoda and Röver, 2001), language proficiency (Bardovi-Harlig and Dörnyei, 1998), motivation (Chiravate, 2012), classroom instruction, length of residence, and L2 community attitudes (Yang and Ren, 2020) are the contributing factors to the different levels of pragmatic awareness. However, the relationship between L2 motivation and pragmatic awareness has been largely underexplored (Yang, 2022). It is necessary to explore the impact of individual differences (such as SES and L2 motivation) on the pragmatic awareness of second language learners (Yang and Ren, 2020; Lv et al., 2021).

In exploring the relationship between SL/FL learning motivation and pragmatic awareness, researchers have found that motivation may play a significant role in students’ pragmatic awareness development in SL/FL learning. Specifically, highly motivated learners tend to exhibit higher levels of pragmatic awareness, that is, succeeding more in recognizing pragmatic inappropriateness or errors than less motivated learners (Chiravate, 2012; Yamato et al., 2013; Yang and Ren, 2020). Among the few studies investigating the impact of L2 learning motivation on pragmatic awareness, Yang and Ren (2020) research has made a significant contribution to this study, as they discovered the significant predictive role of attitudes toward the L2 community, attitudes toward learning English and intended learning efforts on Chinese EFL learners’ pragmatic awareness level. Besides, intrinsic motivation (like ideal L2 self) and communication-oriented motivation (attitudes toward the L2 community) were found to be more closely associated with pragmalinguisitic awareness (Yamato et al., 2013; Takahashi, 2015). However, the existing research design could be further improved in terms of the multifacetedness of motivational variables and group diversity (Botes et al., 2020; Yang and Ren, 2020). Thus, the present study took five motivational variables, that is, ideal L2 self, ought-to L2 self, attitudes toward L2 community, attitudes toward learning English, and intended learning efforts into consideration and invited EFL students from different universities and majors to investigate the predictive effect of motivation on L2 pragmatic awareness.

*H2:* Motivational variables positively predict EFL learners’ PA.

### 2.4 SES, motivation, and PA

There is a relative lack of empirical research on how SES affects pragmatic awareness of foreign languages. On the one hand, research in first language learning has provided preliminary evidence for this study, demonstrating that due to the disadvantage of in-home educational resources (Pace et al., 2017), learners with low SES consistently lag behind their more affluent peers in first language pragmatic development (Pace et al., 2017; Fannin et al., 2018; Qasem et al., 2022). Compared with first language learning, foreign language learning may be more closely associated with learning resources due to the lack of access to the daily linguistic environment for foreign language learners (Bardovi-Harlig and Hartford, 1996; Kasper, 1997; Yang and Ren, 2020). Therefore, this study could reasonably infer that SES significantly affects FL learners’ pragmatic awareness. On the other hand, in FL learning, learners’ SES strengths will help them to be more motivated and achieve higher language proficiency (Butler, 2014; Butler and Le, 2018; Ghorbani and Golparvar, 2020; Lee and Lee, 2023) and finally contribute to their FL pragmatic awareness level (Schauer, 2009; Xu et al., 2009; Yang and Ren, 2020). As mentioned previously, the effect of SES on FL/L2 learning outcomes is considered to be indirect and mediated by other variables. It is logical to further assume that the effect of SES on FL pragmatic awareness is at least partially due to motivation (Huang et al., 2018). However, there is no research investigating the association between SES and FL pragmatic awareness in a single model, let alone further exploring the mediating role of motivation between them. It is quite illuminating to examine the impact of SES on FL learners’ pragmatic awareness and the exact influence pathways.

*H3:* SES positively and directly predicts EFL learners’ PA.

*H4:* Motivational variables significantly mediate the relationship between SES and EFL learners’ PA.

### 2.5 The present study

Based on the literature review, the present study aimed to investigate the relationship among SES, motivation, and pragmatic awareness in EFL learning, particularly the mediating effect of motivation. The result of this study can help educators and teachers design intervention programs to improve students’ pragmatic performance, narrow the learning gap caused by SES, and further promote education equity, which is of great practical significance. The current study sought to address the following questions:

*RQ1:* Is there a direct effect of EFL students’ SES on their motivation?

*RQ2:* Is there a direct effect of EFL students’ motivation on their pragmatic awareness?

*RQ3:* Is there a direct effect of EFL students’ SES on their pragmatic awareness?

*RQ4:* Does EFL students’ learning motivation significantly mediate the relationship between their SES and pragmatic awareness?

## 3 Research design

### 3.1 Participants and procedures

The present study aimed to investigate the relationship between SES and PA, as well as the mediating role of English learning motivation, by using quantitative research methods. Instruments were set to measure students’ SES, motivation, and pragmatic awareness levels. Adopting the convenience sampling method, a questionnaire survey on Chinese EFL learners was conducted. Having obtained permission to conduct the study and use the data only for research purposes, the author of this study contacted university teachers who were asked to forward the questionnaire link to their students. The questionnaires of this study were published through an online survey platform named Wenjuanxing. The teachers introduced the purpose and procedures of the study to all potential participants, and they could voluntarily choose whether to respond to the questionnaires. Excluding questionnaires from participants who submitted incomplete, duplicate, or blank questionnaires and responses of less than 3 min (the minimum time to complete whole questionnaires), the present study collected 292 valid questionnaires voluntarily answered by Chinese EFL students. Participants come from three universities in Beijing, three universities in Hebei, one university in Yunnan, and one university in Guizhou, with a total including 82 male students (28.1%) and 210 female students (71.9%), 87 freshmen (29.8%), 117 sophomores (40.1%), 39 junior students (13.4%), 9 senior students (3.1%), and 40 master students (13.7%), among which 99 students major in foreign language (33.9%), 60 students major in economics and management (20.5%), 51 students major in arts (17.5%), and 82 major in science and engineering (28.1%) (Table 1). Considering the stratification and diversity of the sample, the influence of sampling bias on the generalizability of findings can be greatly reduced.

**Table 1:** Demographic information.

Measure	Item	Frequency	Percentage (%)
Gender	Male	82	28.1
	Female	210	71.9
Grade	Freshman	87	29.8
	Sophomore	117	40.1
	Junior	39	13.4
	Senior	9	3.1
	Postgraduates	40	13.7
Major	Foreign Language	99	33.9
	Economics and Management	60	20.5
	Arts	51	17.5
	Science and Engineering	82	28.1
Total		292	100

### 3.2 Instruments

#### 3.2.1 SES scale

The measurement of SES was adapted from Zheng and Mei (2021) study. Specifically, students’ SES was computed based on their parental educational level (maternal and paternal educational levels), parental occupation (maternal and paternal occupation), and family annual income. A minor adjustment was made to make the scale more appropriate in Chinese. Following Zheng and Mei (2021) study, the student’s final SES score was calculated through the formula “SES score = Education level*0.384 + Occupation*0.371 + Family annual income*0.348″. The scale has a good internal consistency (Cronbach’s *α* = 0.750).

#### 3.2.2 Motivation scale

This motivational questionnaire attempted to measure the following constructs, i.e., ideal L2 self, ought-to self, attitudes toward the L2 community, attitudes toward learning English, and intended learning efforts. All items were answered on a 6-point Likert scale ranging from 1 (strongly disagree) to 6 (strongly agree).

Ideal L2 self and ought-to L2 self scales were derived and adapted from Taguchi et al. (2009) and Papi (2010). Attitudes toward the L2 community, attitudes toward learning English, and intended learning efforts scales were adopted from Yang and Ren (2020). Two independent Chinese-English bilingual teachers obtained the Chinese version of the questionnaire using translation and back-translation methods. The ideal L2 self scale consists of five items: “I can imagine myself speaking English as if I were a native speaker of English.” The ought-to L2 self consists of six items: “Studying English is important to me because other people will respect me more if I have knowledge of English.” Attitudes toward the L2 community scale consist of three items: “I like to travel to English-speaking countries.” The adapted attitudes toward learning English scale consists of two items: “I find learning English interesting.” The intended learning efforts scale includes three items: “I would like to spend lots of time studying English.” Each scale has an ideal reliability (Cronbach’s α = 0.904, 0.911, 0.910, 0.963, 0.900).

#### 3.2.3 Pragmatic awareness test

An appropriateness judgment task (AJT) was used to assess students’ pragmatic awareness. Adopted from Yang and Ren (2020) and Bardovi-Harlig and Dörnyei (1998) studies, the adapted task consists of seven short conversations (e.g., Peter needs directions to the library). He asks another student. A: Hi. P: Hi. P: Tell me how to get to the library, of which four were pragmatically inappropriate, and three were appropriate (controls). Pragmatically inappropriate and appropriate items were developed from responses of nontarget-like learners and native speakers, respectively, both of which were widely accepted and applied in assessing learners’ pragmatic awareness (Yang and Ren, 2020), and a detailed development process of AJT could be seen from Bardovi-Harlig and Dörnyei (1998) study. Learners were asked to assess the appropriateness of the last sentence of each item on a 6-point Likert scale ranging from 1 (strongly inappropriate) to 6 (strongly appropriate). The four pragmatically inappropriate items’ scores were reversed, so the full score of AJT is 42, and the higher the learners’ scores, the higher their pragmatic awareness. The Cronbach’s α was 0.611, indicating an acceptable internal consistency of AJT.

### 3.3 Data analysis

In this study, SPSS 23.0 was used to conduct data standardization, confirmatory factor analysis, common method deviation test, and correlation analysis. Harman’s single-factor test confirmed that there was no significant common method bias in the current study (Podsakoff et al., 2003). Then, Smart PLS 3.0 was utilized to perform the data analysis based on the partial least squares (PLS) method, which could estimate complex models with many latent and manifest variables and is suitable for exploratory studies where the relationship between measures has not been explored. The internal consistency, indicator reliability, convergent validity, and discriminant validity of the measurement model were evaluated first. In this study, the Cronbach’s alpha (CA) of variables ranged from 0.750 to 0.963, greater than 0.7. The composite reliability (CR) ranged from 0.858 to 0.982 (Table 2), greater than 0.7, indicating a good internal consistency (Hair et al., 2019). The Average Variance Extracted (AVE) value is between 0.650 and 0.964 (Table 2), greater than 0.5, explaining a good convergent validity (Fornell and Larcker, 1981). The discriminant validity of the scale was tested through the Fornell-Larcker criterion and the Heterotrait-Monotrait Ratio (HTMT). According to Table 3, the square roots of AVE values are higher than the correlations between constructs (Fornell and Larcker, 1981), and HTMT values are all under 0.85, further proving the discriminant validity of scales (Clark and Watson, 1995). Then, multiple regression analysis was conducted to verify the causal relationship between SES, motivation, and pragmatic awareness, as well as the mediating role of motivational variables, based on the coefficient of determination (*R*^2^), path coefficients, effect size (*f*^2^), and predictive relevance (Q^2^).

**Table 2:** Results for the measurement model.

	Cronbach’s Alpha	CR	AVE
SES	0.750	0.858	0.670
ILS	0.904	0.926	0.718
OLS	0.911	0.914	0.650
ATLC	0.910	0.943	0.847
ATLE	0.963	0.982	0.964
ILE	0.900	0.926	0.760

SES, socioeconomic status; ILS, ideal L2 self; OLS, ought-to L2 self; ATLC, attitudes toward the L2 community; ATLE, attitudes toward learning English; ILE, intended learning efforts.

**Table 3:** Fornell-Larcker criterion and HTMT.

	Model 1			Model 2			Model 3			Model 4			Model 5		
Fornell-Larcker criterion															
	SES	ILS	PA	SES	OLS	PA	SES	ATLC	PA	SES	ATLE	PA	SES	ILE	PA
SES	0.817			0.811			0.815			0.813			0.812		
CMV	0.183	0.847		−0.035	0.805		0.260	0.921		0.210	0.982		0.143	0.871	
PA	0.164	0.253	1	0.169	−0.175	1	0.167	0.233	1	0.168	0.196	1	0.169	0.162	1
HTMT															
SES															
CMV	0.204			0.077			0.306			0.238			0.143		
PA	0.186	0.236		0.186	0.148		0.186	0.243		0.186	0.199		0.186	0.151	

CMV, corresponding mediating variable; SES, socioeconomic status; ILS, ideal L2 self; OLS, ought-to L2 self; ATLC, attitudes toward the L2 community; ATLE, attitudes toward learning English; ILE, intended learning efforts.

## 4 Results

### 4.1 Correlation analysis

The Pearson correlation analysis tested the relationship among learners’ SES, motivation, and pragmatic awareness. The results showed that learners’ SES was significantly and positively correlated with their ideal L2 self, attitudes toward the L2 community, attitude toward English, intended learning efforts, and pragmatic awareness, and is not related to ought-to L2 self. Besides, among five motivational variables, ideal L2 self, attitudes toward the L2 community, attitude toward English, and intended learning efforts were significantly and positively correlated with learners’ pragmatic awareness. There was no significant correlation to be found between learners’ ought-to self and pragmatic awareness (Table 4).

**Table 4:** Results of Pearson correlation coefficient.

	SES	ILS	OLS	ATLC	ATLE	ILE	PA
SES	1						
ILS	0.168**	1					
OLS	0.09	0.287**	1				
ATLC	0.253**	0.635**	0.216**	1			
ATLE	0.202**	0.649**	0.231**	0.588**	1		
ILE	0.118*	0.513**	0.270**	0.367**	0.691**	1	
PA	0.161**	0.225**	−0.113	0.232**	0.195**	0.144*	1

* *p* < 0.05,** *p* < 0.01. SES, socioeconomic status; ILS, ideal L2 self; OLS, ought-to L2 self; ATLC, attitudes toward the L2 community; ATLE, attitudes toward learning English; ILE, intended learning efforts; PA, pragmatic awareness.

### 4.2 The direct effect of EFL students’ SES on their motivation

SES was found to have a significantly positive effect on students’ ideal L2 self, attitudes toward the L2 community, and attitudes toward learning English, and no effect on students’ ought-to L2 self and intended learning efforts.

### 4.3 The direct effect of EFL students’ motivation on their PA

All five motivational variables significantly predict EFL students’ pragmatic awareness, among which ideal L2 self, attitudes toward L2 community, attitudes toward learning English, and intended learning efforts positively predict learners’ pragmatic awareness while ought-to L2 self negatively predicts it.

### 4.4 The direct effect of EFL students’ SES on their PA

SEM was conducted to test the five proposed models, respectively. As Table 5 shows, the regressive analysis indicated that SES directly affected EFL learners’ pragmatic awareness. SES has shown a significant and positive effect on English learners’ pragmatic awareness level in all five proposed models. However, the effect size is weak (*f*^2^ is around 0.02, the low threshold value indicating the predictor’s effect). The proportion of this direct effect to the total effect varies among the five models, ranging from 67.7 to 100%. It could be concluded that SES accounts for a certain portion of the discrepancies among English learners’ pragmatic awareness levels.

**Table 5:** The mediating role of motivational variables.

Model	Mediating path	DE	IE	TE	VAF	Pathway	*R* ^2^	*f* ^2^	Q^2^
1	SES → ILS → PA	0.119*	0.042*	0.161**	0.261	SES → ILS	0.033	0.034	0.020
						SES → PA	0.078	0.015	0.061
						ILS → PA		0.056	
2	SES → OLS → PA	0.157*	0.004	0.161**	0	SES → OLS	0.001	0.001	−0.001
						SES → PA	0.055	0.026	0.038
						OLS → PA		0.031	
3	SES → ATLC→PA	0.109*	0.052*	0.161**	0.323	SES → ATLC	0.064	0.069	0.052
						SES → PA	0.066	0.012	0.052
						ATLC→PA		0.042	
4	SES → ATLE→PA	0.127*	0.034*	0.161**	0.211	SES → ATLE	0.041	0.043	0.031
						SES → PA	0.054	0.016	0.032
						ATLE→PA		0.029	
5	SES → ILE → PA	0.142**	0.019	0.161**	0	SES → ILE	0.019	0.019	0.008
						SES → PA	0.046	0.021	0.020
						ILE → PA		0.021	

DE, direct effect; IE, indirect effect; TE, total effect; SES, socioeconomic status; ILS, ideal L2 self; OLS, ought-to L2 self; ATLC, attitudes toward the L2 community; ATLE, attitudes toward learning English; ILE, intended learning efforts; PA, pragmatic awareness. *p < 0.05, **p < 0.01 (2-tailed).

### 4.5 The mediating role of EFL students’ motivation between their SES and PA

**Figure 1:**
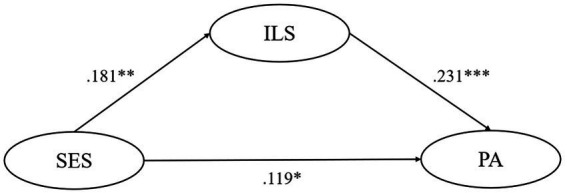
The mediating role of ideal L2 self. * p < 0.05, ** p < 0.01, *** p < 0.001. SES, socioeconomic status; ILS, ideal L2 self; PA, pragmatic awareness.

**Figure 2:**
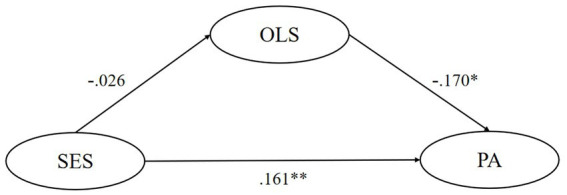
The mediating role of ought-to L2 self. * p < 0.05, ** p < 0.01, *** p < 0.001. SES, socioeconomic status; OLS, ought-to L2 self; PA, pragmatic awareness.

**Figure 3:**
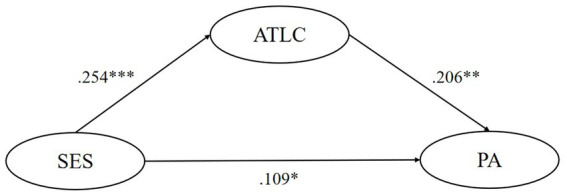
The mediating role of attitudes toward the L2 community. * p < 0.05, ** p < 0.01, *** p < 0.001. SES, socioeconomic status; ATLC, attitudes toward the L2 community; PA, pragmatic awareness.

**Figure 4:**
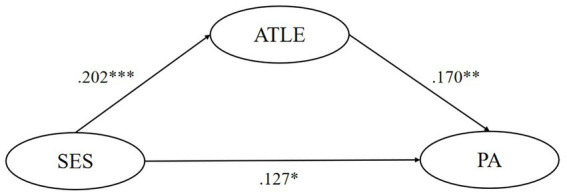
The mediating role of attitudes toward learning English. * p < 0.05, ** p < 0.01, *** p < 0.001. SES, socioeconomic status; ATLE, attitudes toward learning English; PA, pragmatic awareness.

**Figure 5:**
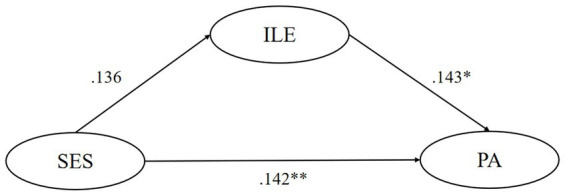
The mediating role of intended learning efforts. * p < 0.05, ** p < 0.01, *** p < 0.001. SES, socioeconomic status; ILE, intended learning efforts; PA, pragmatic awareness.

SEM tested the mediating effect of five motivational variables respectively, finding that the ideal L2 self, attitudes toward the L2 community, and attitudes toward learning English play significant mediating roles in the relationship between their SES and pragmatic awareness. Though ought-to L2 self and intended learning effort were found to significantly and negatively predict pragmatic awareness, their mediating role is insignificant. In model 1, the mediating effect of ideal L2 self accounts for 26.1% of the total effect, and this model could predict 7.8% of learners’ pragmatic awareness variance. In model 3, the mediating effect of attitudes toward the L2 community accounts for 32.3% of the total effect, and this model could predict 6.6% of learners’ pragmatic awareness variance. In model 4, the mediating effect of attitudes toward learning English accounts for 21.1% of the total effect, and this model could predict 5.4% of learners’ pragmatic awareness variance. All statistically significant causal relationships found above have substantial but slightly weak explanatory power (0.19 > *R*^2^ > 0) and effect size (*f*^2^ near or over 0.02) (Cohen, 1988; Chin, 1998). Besides, all of the five models have good predictive relevance (Q^2^ > 0) except for the predictive effect of SES on ought-to self (Q^2^ = −0.001 < 0) (Geisser, 1975) (Figures 1–5).

## 5 Discussion

### 5.1 The influence of SES on ought-to L2 self and intended learning efforts

There was no significant relationship found between SES and ought-to L2 self and intended learning efforts in the present study, revealing that SES does not have a predictive effect on the state that English learners achieve to satisfy significant others and the efforts they intended to apply to English learning, which was against H1. This result may be attributed to the compulsoriness and popularity of the Chinese university English curriculum (Lamb, 2012). In Chinese universities, English courses are generally offered and taken as compulsory subjects. Students develop instrumental motivation toward English learning to meet the expectations of parents and teachers or to avoid negative outcomes like failing exams or letting others down. That is, once students take English courses and exams, they have strong, oblationary feelings to put effort into making it regardless of their family background.

### 5.2 The influence of ought-to L2 self and intended learning efforts on PA

The predictive role of ought-to L2 self and intended learning efforts on English learners’ pragmatic awareness was revealed in the present study. Specifically, a negative correlation was found between ought-to L2 self and pragmatic awareness, which was against H2. That is to say, the stronger a student’s ought-to L2 self is, the less conducive it is to their development of English pragmatic awareness, which is similar to previous results documenting the negative effect of ought-to L2 self on L2 learning (Papi and Teimouri, 2014; Peng, 2015; Feng and Papi, 2020). A possible reason for the negative correlation between ought-to L2 self and pragmatic learning outcomes is the mismatch between Chinese students’ immediate English learning needs and English pragmatic learning (Yang and Ren, 2020). Unlike the ideal L2 self, the ought-to L2 self is more like an instrumental motivation (Lamb, 2012), which primarily focuses on current learning outcomes and how to satisfy others’ expectations (Al-Hoorie, 2018). In the Chinese exam-oriented education system, driven by this extrinsic motivation, students think passing exams and getting teachers’ approval are the most important and urgent issues (Yang and Ren, 2020). College English Test (Band 4 or Band 6) is a well-recognized national English level test among university students in China. It mainly examines writing and reading literacy (Taguchi and Roever, 2017). Under this circumstance, students are more motivated to improve receptive skills such as listening and reading comprehension with focuses on grammatical and vocabulary enrichment, while pragmatic learning, which will not be tested, such as appropriate expressions of daily conversation, has been largely neglected, which finally has a negative effect on EFL learners’ motivation to develop pragmatic awareness. In addition, a positive and significant relationship between intended learning efforts and pragmatic awareness was found, which supported H2 and Yang and Ren (2020) findings that when students are willing to put effort into English learning, they tend to perform well in pragmatic awareness test, and further confirming the significant role of motivation in L2 pragmatic development.

### 5.3 The direct predictive effect of EFL students’ SES on PA

This study investigated the relationship between SES and English pragmatic awareness among Chinese university students. The results showed that students’ SES could positively and directly predict their pragmatic awareness level, which answered our research question 3 and verified H3. The results indicated that EFL students with higher family SES are more likely to notice pragmatic issues and perform better in pragmatic awareness tests. Previous studies have shown the positive effect of SES on children’s pragmatic performance in first language learning (Fannin et al., 2018; Qasem et al., 2022). The current study found this same relationship in the FL learning context. The influence existed, although the effect size was between the low and middle ranges. In FL learning, the target language does not exist in the learner’s direct environment. In the case of insufficient pragmatic contact in class, extracurricular language access appears particularly important for Chinese EFL students to cultivate their pragmatic competence (Yang and Ren, 2020). Therefore, SES has been attached great importance in FL learning since high SES families are more likely to provide students with learning environments and pragmatic resources helpful for their development of communicative abilities, which contributes to their pragmatic awareness (Niezgoda and Röver, 2001; Schauer, 2009; Butler and Le, 2018; Ghorbani and Golparvar, 2020). However, not all parents can provide such a kind of promoting learning environment and resource for their children. Differences in family SES can lead to discrepancies in students’ pragmatic performance. Therefore, exploring the mediating pathways to bridge this gap is even more important.

### 5.4 The mediating effect of motivation between SES and PA

The study further examined the mediating effect of motivational variables between English learners’ SES and pragmatic awareness. The results suggested that the ideal L2 self, attitudes toward the L2 community, and attitudes toward learning English significantly mediate the relationship between SES and pragmatic awareness, indicating that English learners with higher SES levels are more likely to see themselves as fluent English speakers, hold more positive attitudes toward English speaking countries and people and are more interested in learning English, which in turn contribute to their performance in pragmatic awareness test. These findings answered RQ4 and supported H1, H2, and H4. It is worth noticing that these associations are statistically significant but have small explanatory and influential power (Cohen, 1988; Chin, 1998), which may be attributed to some external factors beyond motivation (Schmidt, 1993; Bardovi-Harlig and Dörnyei, 1998; Ren and Han, 2016; Yang and Ren, 2020). According to Dörnyei (2005, 2009), students’ immediate language learning experience may greatly affect their motivation to learn the target language. In Yang and Ren (2020) interviews with Chinese university English learners, they reported some common issues that exist in the Chinese English teaching environment, that is, lack of pragmatics instruction, lack of opportunities to practice pragmatic knowledge, and various pragmatic norms that students find difficult to choose one to follow (Yang and Ren, 2020). That is to say, although students develop motivation to learn English, these factors will, to some degree, hinder them from greatly improving their pragmatic achievement (Yang and Ren, 2020), resulting in these significant but weak influences.

#### 5.4.1 The mediating effect of ideal L2 self between SES and PA

Among three motivational mediating variables, the ideal L2 self contributes more to English learners’ pragmatic awareness. It is reasonable to assume that when socioeconomically privileged English learners hold an optimistic view toward their future success in learning English, they ought to perform better in pragmatic tasks. This result confirmed previous studies supporting that SES positively influences learners’ vision of future success in English learning (Kim and Kim, 2014; Papi and Teimouri, 2014; Khan, 2015). For example, Lamb (2012) and Kormos and Kiddle (2013) found that students from high-class families were more optimistic about their future language competence than those from low-class families. Oyserman and Fryberg (2006) emphasized the importance of a social environment for learners’ future self-guides. Peng (2015) said that learners’ ideal L2 self would be triggered or enhanced by successful and pleasant personal experiences. Students with better SES are more likely to encounter role models during the English learning process. Positive social contact with prestigious people is conducive to their development of an ideal L2 self in English learning (Lamb, 2012). In conclusion, learners with high SES are more capable of learning English in such a context where socializing with English speakers, encountering role models, and participating in international competitions are accessible, tending to imagine themselves becoming fluent language users. This practically orientated mental image makes students more persistent and more focused on the actual use of the target language (Feng and Papi, 2020) and finally contributes to their development of pragmatic awareness (Schmidt, 1993; Bardovi-Harlig and Dörnyei, 1998; Kormos and Kiddle, 2013).

Besides, the mediating effect also showed a positive correlation between ideal L2 self and pragmatic awareness, showing that when EFL learners have a positive attitude toward their future English achievements, they will better assess the appropriateness of English speech acts. Specifically, when students aspire to become capable and proficient English speakers, they are keen to reduce discrepancies between their ideal and present selves and focus on the actual use of the target language, particularly successful communication, such as writing emails and engaging in academic exchanges (Taguchi et al., 2009). In this case, the discrepancies between the ideal and present self may facilitate the development of students’ pragmatic awareness. This conclusion is consistent with previous research that learners with intrinsic motivation and communication-oriented motivation tend to exhibit higher levels of pragma linguistic awareness (Yamato et al., 2013; Takahashi, 2015) but inconsistent with Yang and Ren (2020) research finding no significant correlations between ideal L2 self and English pragmatic awareness. Students’ different interpretations of the ideal self may be a possible reason for inconsistent results. In Yang and Ren (2020) research, the students being interviewed came from a prominent university in China with a prevalent academic atmosphere. Their reported ideal selves were more about their ability to meet immediate academic accomplishments, which led students to focus on listening, reading, and writing literacy rather than practical aspects of English. In contrast, students in the present study came from a wider range of universities. By eliminating the limitation of sample singularity, the obtained results may be closer to objective reality and more referential.

Notably, the ideal L2 self rather than the ought-to L2 self was found to mediate the association between SES and pragmatic awareness. This result is consistent with previous studies based on the L2MSS, demonstrating that the ideal L2 self is a better predictor of learning outcomes than the ought-to L2 self (Al-Hoorie, 2018; Sadoughi et al., 2023). Researchers have found it is ideal L2 self that positively predicted the use of self-regulated learning writing strategies (Xu and Wang, 2022), academic engagement (Sadoughi et al., 2023), and achievement (Dörnyei and Chan, 2013; Al-Hoorie, 2018), rather than ought-to L2 self. These differential results may relate to the promotion focus of the ideal L2 self and the prevention focus of the ought-to L2 self (Dörnyei, 2009). Therefore, we believe that the ideal L2 self is the primary factor affecting EFL learners’ pragmatic awareness and demonstrate the more internalized and active role of the ideal self in motivating students and empowering their L2 learning strength.

#### 5.4.2 The mediating effect of attitudes toward L2 community and attitudes toward learning English between SES and PA

Attitudes toward the L2 community and attitudes toward learning English significantly mediate the relationship between English learners’ SES and their pragmatic awareness level, indicating that students with higher family SES are typically more likely to have a positive and friendly attitude toward English-speaking countries and people there, and tend to be more proactive and engaged in English learning, which would assist them in succeeding in judging English pragmatic appropriateness. Prior research stated that learners from low social class families typically lacked awareness of the importance of English, thus having their future visions weakly correlated with English (Lamb, 2012). There are still some students who have realized the significance of pragmatic learning but put it on hold, suffering from no resources to learn and no urgent pragmatic needs (neither taking pragmatic exams nor necessity to communicate with foreigners) (Yang and Ren, 2020). Under these circumstances, learners rarely envision their future development linked to English, and they are less motivated to learn English, finding themselves lacking interest and effort in English. In contrast, learners from upper-class families, as Kormos and Kiddle (2013) argued, often believe that they will study abroad or be involved in international competition, where English is an indispensable part of their lives. These future communicative needs will drive them to develop pragmatic motivations. It is logical to suppose that when students come from families with high SES, they place themselves on the international stage, are eager to socialize with English speakers and exert themselves to learn English. Previous research has fully demonstrated the positive correlation between students’ SES and English learning motivation (Kormos and Kiddle, 2013; Butler, 2015; Lee and Lee, 2023), and this correlation has become stronger as grades increase. The current study found three motivation variables to be significantly correlated with SES, which to some extent further confirmed the above conclusion among university students. Moreover, this result is confirmed by Yang and Ren (2020) research finding that Chinese students’ attitudes toward the L2 community are significantly related to their performance in pragmatic awareness test, as well as to some degree conforming to Schumann (1986)acculturation model, which holds that the psychological distance between learners and the target community has an impact on their L2 learning.

## 6 Conclusions, implications, limitations, and suggestions

The present study examined the relationship between university EFL students’ socioeconomic status and pragmatic awareness with the mediating role of English learning motivation, which demonstrates some motivation variables as pathways in which socio-economic inequality can contribute to students’ PA gap. The theoretical contribution of the study lies in the mediating effect of an ideal self, attitudes toward the L2 community, and attitudes toward learning English between EFL students’ family socio-economic background and their appropriate judgment capacities concerning pragmatics. The relationship between socioeconomic status and pragmatic acquisition was also expanded from the first language field to the foreign language learning area and from younger learners such as primary and secondary school students to university students. In terms of practical instruction, with specific clarification of the association between the different variables, it is important to design and develop proper interventions to enhance students’ pragmatic judgment abilities. Hopefully, the findings will encourage English educators to consider students’ individual differences in pragmatic instruction and provide the foundation for effective intervention in future instruction.

Based on research findings, the study proposed some substantial implications for educators. Teachers should recognize learners’ diverse SES backgrounds and needs in class, as well as the different impacts of motivational variables on English pragmatic awareness. Classroom instruction should include stimulating learners’ pragmatic interests and needs and providing learners with pragmatic help and practice opportunities. Differentiated teaching strategies (Xu and Feng, 2024) are expected to be implemented if human and material resources permit. On the one hand, the generally current instruction situation that undermines students’ pragmatic motivation should be addressed. Incorporating pragmatic instruction into EFL teaching curricula is of the essence (Qin et al., 2024; Yang and Ren, 2020). For example, the design of textual and oral practices that reflect real-life situations in both academic and daily life can allow learners to develop and apply their pragmatic awareness in a specific context (Qin et al., 2024). In addition, increasing learners’ knowledge of the effect of their L1 on L2 pragmatic uses and making contrastive analyses are conducive to their notice and judgment of L2 pragmatic inappropriateness (Qin et al., 2024). Besides, sample conversations of native English speakers in specific contexts, such as academic conferences and emails, can serve as effective learning resources for learners to cultivate language intuition and understand the norms of the target language (Qin et al., 2024).

On the other hand, cultivating learners’ pragmatically related motivation, that is ideal L2 self, attitudes toward the L2 community, and attitudes toward learning English, will help. By providing positive feedback and constructive suggestions, teachers can assist students in establishing their vision of future success in EFL learning, facilitating their development of pragmatic awareness (Yang and Ren, 2020). An immersive language learning environment, that is, making learners more exposed to the English environment, can increase learners’ interest in English learning and the L2 community. The set of pragmatic instruction and practice mentioned above, as well as the inclusion of English movies, music, blogs, and other elements in curricula, are effective measures to increase learners’ contact with the target language. Notably, the addition of pragmatic instruction and exercise to the English curriculum might be conducive to the pragmatic learning of students driven by ought-to L2 self. They might attach importance to pragmatic learning because it has become part of their curriculum and tests (Yang and Ren, 2020).

This study also has some limitations. First, adopting the convenience sampling method in preliminary research with a large sample is appropriate to quickly conclude and lay the groundwork for the follow-up study. The appropriateness of the sampling method and stratified and diversified sample guarantee that the results of this study have a certain generalisability among Chinese EFL students. However, what should be acknowledged is that this study still has some sample limitations in terms of region and gender. Future studies ought to supplement qualitative material and collect more comprehensive and diverse data to further verify and enhance the strength and applicability of these conclusions in other populations and contexts. Secondly, this study adopted self-reported measurement to assess students’ motivation levels. Discrepancies may exist between their actual conditions and reported conditions. Therefore, future studies should consider more measurements, such as interviews or others’ evaluations, to obtain students’ most authentic motivation level. In addition, this study examined EFL learners’ motivation based on L2MSS theory, which has been well-established in SLA but has also been criticized for overlooking students’ important motivation forces, resulting in the study’s shortcomings in revealing students’ motivation beyond language learning goals. Hence, future studies should consider more novel and comprehensive motivational frameworks to compensate for this deficiency. Last but not least, given that students’ L2 motivation is dynamic, longitudinal investigations are needed to reveal the dynamic variation of their motivation during a period of English learning.

## Data Availability

The datasets presented in this article are not readily available because part of the data was involved in other unpublished studies. Requests to access the datasets should be directed to Yuqing Chen, neuwy2021@163.com.
